# Human liver rate-limiting enzymes influence metabolic flux via branch points and inhibitors

**DOI:** 10.1186/1471-2164-10-S3-S31

**Published:** 2009-12-03

**Authors:** Min Zhao, Hong Qu

**Affiliations:** 1 Center for Bioinformatics, National Laboratory of Protein Engineering and Plant Genetic Engineering, College of Life Sciences, Peking University, Beijing, 100871, PR China

## Abstract

**Background:**

Rate-limiting enzymes, because of their relatively low velocity, are believed to influence metabolic flux in pathways. To investigate their regulatory role in metabolic networks, we look at the global organization and interactions between rate-limiting enzymes and compounds such as branch point metabolites and enzyme inhibitors in human liver.

**Results:**

Based on 96 rate-limiting enzymes and 132 branch point compounds from human liver, we found that rate-limiting enzymes surrounded 76.5% of branch points. In a compound conversion network from human liver, the 128 branch points involved showed a dramatically higher average degree, betweenness centrality and closeness centrality as a whole. Nearly half of the *in vivo *inhibitors were products of rate-limiting enzymes, and covered 75.34% of the inhibited targets in metabolic inhibitory networks.

**Conclusion:**

From global topological organization, rate-limiting enzymes as a whole surround most of the branch points; so they can influence the flux through branch points. Since nearly half of the *in vivo *enzyme inhibitors are produced by rate-limiting enzymes in human liver, these enzymes can initiate inhibitory regulation and then influence metabolic flux through their natural products.

## Background

The liver is the largest organ to metabolize most compounds in the body [1]. The interaction between biochemical compounds and enzymes is the fundamental mechanism for dynamically adapting to a variety of environmental or *in vivo *conditions [2,3]. In recent years rapid development of high-throughput proteomics technology, such as mass spectrometry, provide the opportunity to investigate metabolic flux at a systematic level [4-6].

Before high-throughput flux analysis, many concepts were proposed to explain the dynamic flux control in individual pathways, including rate-limiting enzymes and branch point compounds [7-9]. All these concepts focus on flux control coefficients of an isolated enzyme or compound in a pathway. According to the rate-limiting concept, at least one reaction far from equilibrium is catalyzed by rate-limiting enzymes at a relatively lower velocity than other enzymes in the same pathway. The rate of this reaction is not determined by substrate concentration, but only by the activities of these enzymes. At the compound level, compounds located at branch points are described as essential molecules that influence flux [10-13], and the kinetic properties of these branch points confirm their role in directly determining the flux rate [12].

Despite their importance in flux control, the global organization and interactions among rate-limiting enzymes and branch points have not been explored to date. Several rate-limiting enzymes were reported to interact with branch points, including isocitrate dehydrogenase and inosine 5'-monophosphate dehydrogenase [14-16]. Since many small-scale studies of rate-limiting enzymes and branch points are scattered throughout the literature, it has been difficult, so far, to investigate the global interactions between rate-limiting enzymes and branch points.

Studying an individual pathway is not sufficient to identify the properties of global organization. The extent to which flux is controlled by rate-limiting enzymes in an individual pathway is not the most important feature at the systematic level [17]. The crucial question at the systematic level is, whether rate-limiting enzymes as a whole can respond to regulatory signals and trigger subsequent metabolic events [18]. To do systematic analysis of rate-limiting enzymes, we manually curated 383 rate-limiting enzymes in five organisms, human, rat, mouse, yeast and *E. coli *and constructed the first literature-based Rate-Limiting Enzyme database (RLEdb) [19].

Enzyme inhibition is a short-term regulatory interaction between compounds and enzymes. Thousands of enzyme inhibitors have been used *in vitro *and *in vivo *to study metabolic enzyme properties [20]. Using such data, biochemists can set objective functions to estimate the regulatory effectiveness of inhibitors at the pathway level [21,22]. At the genome level, although enzyme inhibition and activation networks have been studied [23], the focus was on global properties of their metabolic regulatory networks and the chemical structures of inhibitors. The relations between inhibitors and essential enzymes for flux control, such as rate-limiting enzymes, have not yet been studied.

Here, we made an extensive collection of rate-limiting enzymes, branch points and inhibitors from human liver and attempted to answer basic questions about the global organization and interactions between these molecules. How many rate-limiting enzymes are located before and after branch points? How do they influence flux together and transmit regulatory signals? How many enzymes can be regulated by *in vivo *inhibitors? What kind of enzymes can produce *in vivo *inhibitors? What are the ideal inhibited targets able to accept and transmit metabolic signals among different pathways?

## Results

Our study is based on five distinct datasets: (i) 687 metabolic enzymes of human liver compiled from the HPRD database [24] and KEGG ligand database [25,26]; (ii) all 1033 products of these 687 enzymes; (iii) 96 liver rate-limiting enzymes manually collected from 2682 PubMed abstracts; (iv) 132 branch points curated from KEGG pathway maps; a branch point is defined to be any compound connected with three or more enzymes, at least one of which should be able to produce that compound and one that can consume it; and (v) 202 enzyme inhibitors collected from the BRENDA database [20]. Based on these datasets, we constructed two types of metabolic network. One was the compound conversion network taken from the KEGG ligand-rpair database [25,26]; the other was the inhibitory network compiled from the BRENDA database [20].

### Rate-limiting enzymes surround 76.5% of the branch point compounds in total

**Figure 1:**
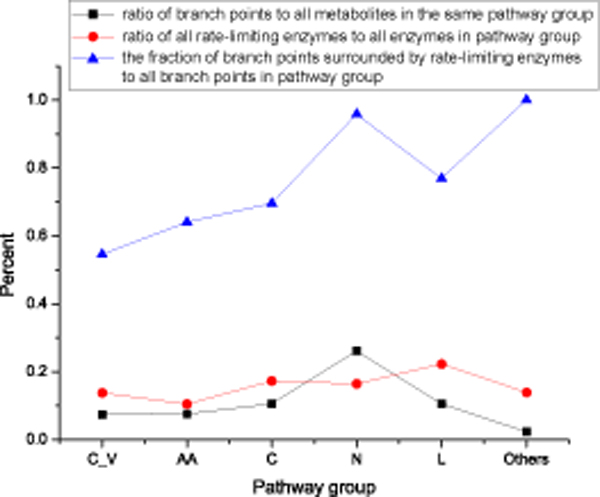
**Statistics for branch points and rate-limiting enzymes**. Black represents the ratio of branch points to all metabolites for each pathway group, red represents the ratio of rate-limiting enzymes to all metabolic enzymes for each pathway group, and blue is the fraction of branch points surrounded by rate-limiting enzymes to all branch points for each pathway group. The pathway names on the x-axis are: C_V (metabolism of cofactors and vitamins), AA (amino acid metabolism), C (carbohydrate metabolism), N (nucleotide metabolism), L (lipid metabolism) and Others (other metabolism pathways).

To survey the pathway distribution of all rate-limiting enzymes and branch points, we classified all the rate-limiting enzymes into six pathway groups according to the KEGG hierarchy pathway annotation: Carbohydrate metabolism, Lipid metabolism, Nucleotide metabolism, Amino acid metabolism, Cofactor and vitamin metabolism and Others metabolism. On average, both branch points and rate-limiting enzymes made up less than 20% of the compounds and enzymes in human liver (Figure 1). Different pathway groups showed different topological structures in terms of the composition of branch points. The proportion of branch points in nucleotide metabolism was a little higher than in the other pathway groups. The fraction of rate-limiting enzymes was similar in all pathway groups. This meant that the proportion of the flux control point, such as a rate-limiting step, was almost the same.

From global topological organization, a large proportion of branch points in each pathway group were surrounded by rate-limiting enzymes (Figure 1). In total, 76.5% of the branch points were surrounded by 60 rate-limiting enzymes. Since the reactions surrounding a branch step can be used to modulate metabolic flux, the enzymes surrounding branch points can influence the branch flux in the pathway.

Furthermore, to survey the topological relations of all rate-limiting enzymes and branch points in the different types of pathways, we annotated all the rate-limiting enzymes into 4 classes according to functional hierarchies and ontologies of KEGG BRITE: central, catabolic, anabolic and energetic pathways. In the dataset of human liver, no significant differences were found between the number of rate-limiting enzymes located before and after branch points (Table 1). Also, no evident difference was found between the numbers of branch points as substrates of rate-limiting enzymes and branch points as products of rate-limiting enzymes. In addition, the distribution of topologic relations of rate-limiting enzymes and branch points in the 4 classes of enzymes also did not differ much (Table 1).

**Table 1:** Before-after relations between branch points and rate-limiting enzymes.

	RL_after_BP	RL_before_BP	Substrate_of_RL	Product_of_RL
Human	49	45	78	67
Liver				
Central	3	4	6	7
Anabolic	18	19	40	35
Catabolic	14	12	26	24
Energetic	1	2	3	2

RL_after_BP column records the number of rate-limiting enzymes which occur directly after certain branch points. RL_before_BP column records the number of rate-limiting enzymes which occur directly before certain branch points. Substrate_of_RL column records the number of branch points, which are substrates of certain rate-limiting enzymes. Product_of_RL column records the number of branch points, which are products of certain rate-limiting enzymes. The human liver row is the statistic for the entire human liver dataset, and the other four rows are the statistics for the enzymes and branch points from central, anabolic, catabolic and energetic pathways.

### Branch points show high degree, betweenness centrality and closeness centrality in compound conversion network

**Figure 2:**
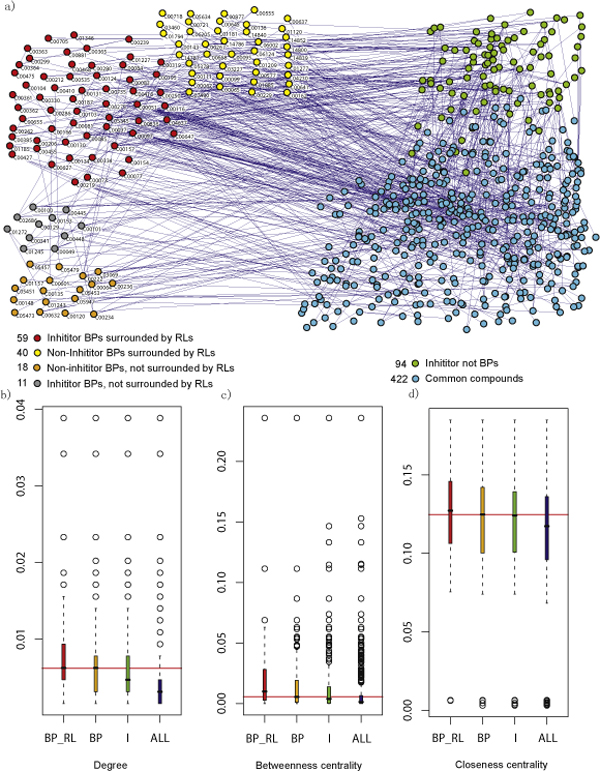
**Characteristics of branch point metabolites in compound conversion network**. (a) Compound conversion network in human liver. BPs represent branch points. RLs represent rate-limiting enzymes. The inhibitor BPs represent that the branch points are also enzyme inhibitors. The blue lines between pairs of nodes represent the conversion relation between them. (b, c, d) Boxplots are for degree, betweenness centrality and closeness centrality of branch points and all the metabolites in the compound conversion network. In each boxplot, the red bar represents the average degree, betweenness centrality and closeness centrality for 59 inhibitor branch points surrounded by rate-limiting enzymes; the orange bar represents the average degree, betweenness centrality and closeness centrality for all 128 branch points; the green bar represents the average degree, betweenness centrality and closeness centrality for all 164 inhibitors, and the blue bar represents the average degree, betweenness centrality and closeness centrality for all 644 compounds in the compound conversion network.

To study the function of branch points in human liver, we constructed an undirected compound conversion network by combining information on all the rpair entries in human liver from the KEGG database. Each rpair entry records a pair of compounds which are converted directly via certain enzymes. In this network, a node represented an individual compound produced by any enzyme. Two compounds were connected if they shared a rpair entry and the enzymes to convert the pair of compounds also occurred in human liver. We assumed that the reactions to convert each pair of compounds were reversible and therefore the compound-compound relation in the network was undirected. The compound conversion network contained 644 nodes (the remaining 389 compounds did not convert to other liver compounds according to rpair data) and 890 links (Figure 2a). Among the 644 compounds, there were 128 branch points (the remaining 4 branch points did not convert to other liver compounds according to rpair data) and 164 enzyme inhibitors (the other 58 inhibitors did not convert to other liver compounds according to rpair data). The degree of all nodes tended to follow a power law distribution *P*(*k*)~*k*^-*r*^, where *P*(*k*) was the probability that a node has *k *connections and *r *was an exponent with an estimated value of 2.3298 for the compound conversion network shown here (Figure 2a). This indicates that most compounds in our network were sparsely connected while only some had very high degree. Therefore, our metabolite conversion network is a typical scale-free network and its degrees follow a power-law distribution [27-30].

To test which important topological roles are executed by branch points in the compound conversion network, degree, betweenness centrality and closeness centrality of each node were calculated using Pajek [31]. The degree, the number of connections of each node, is a local property. The higher the degree, the higher the probability of this node to convert to other compounds in this network. By contrast, the betweenness centrality measures how frequently a node appears on all shortest pathways between two other nodes. And closeness centrality measures how many steps it requires to connect to other vertices from a given vertex. Closeness is preferred in network analysis to mean shortest-path length, as it gives higher values to more central vertices, and so is usually positively associated with other measures such as degree.

Statistical significance analyses of the average degree, betweenness centrality and closeness centrality of branch points against all the metabolites in human liver were performed. The averages of the three types of centrality from branch points were higher than those of all metabolites in human liver (unequal 2-tailed t-test, P-value < 0.001; Figure 2bcd). Intuitively, the 128 branch points must have high average degrees compared with the entire population of 644 metabolic compounds, since we defined the branch points as having a higher local connection number than common compounds. The higher average betweenness centrality and closeness centrality confirm the central role of branch points in metabolite conversion. Higher average betweenness centrality indicates that branch points are more likely to be located in the shortest pathways between two other compounds as a whole. Higher average closeness centrality indicates that branch points easily reach other compounds in shorter steps. Similar statistical significance analyses for all 164 inhibitors were also performed. The averages of the three types of centrality from inhibitors were also higher than those of all metabolites.

### Nearly half of the inhibitors are the products of rate-limiting enzymes, and they inhibit most targets *in vivo*

According to our *in vivo *inhibitor annotation, nearly half of the inhibitors are products of rate-limiting enzymes in human liver (96 versus 204), and they can potentially inhibit most of their *in vivo *targets. First, enzyme-enzyme relationships can be established for two enzymes if the product of one is the inhibitor of the other. The inhibitor initiator is the enzyme that provides the inhibitor in each enzyme pair; the inhibitor target is the other inhibited enzyme.

**Figure 3:**
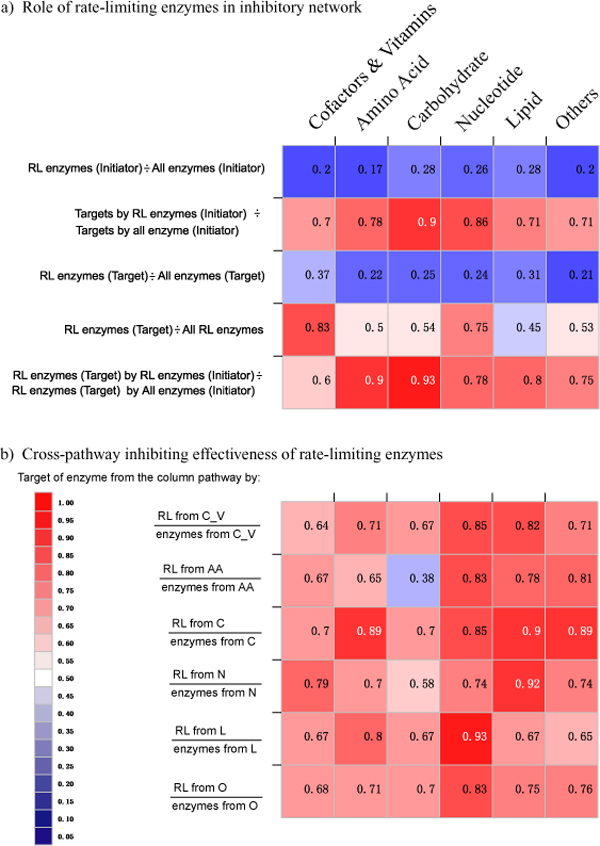
**Characteristics of rate-limiting enzymes in inhibitory network**. (a) Color-grid for the role of rate-limiting enzymes in the inhibitory network as inhibiting initiators and targets. (b) Inhibitory efficiencies of rate-limiting enzymes as inhibitor initiators in inhibitory networks pairwise among six pathway groups. For each cell, the ratio represents the inhibited enzymes in the column pathway group by the products of rate-limiting enzymes from the row pathway group to the inhibited enzymes in the column pathway by the products of all metabolic enzymes from the row pathway group. The pathway names on left are: C_V (metabolism of cofactors and vitamins), AA (amino acid metabolism), C (carbohydrate metabolism), N (nucleotide metabolism), L (lipid metabolism) and O (other metabolism pathways).

Figure 3 illustrates the characteristics of rate-limiting enzymes in inhibitory network. In Figure 3a, where RL enzymes (Initiator) represent the number of rate-limiting enzymes whose products are inhibitors, All enzymes (Initiator) represent the number of all metabolic enzymes whose products are inhibitors, Targets by RL enzymes (Initiator) represent the number of target enzymes inhibited by rate-limiting enzymes, Targets by all enzymes (Initiator) represent the number of target enzymes inhibited by all the metabolic enzymes, RL enzymes (Target) represent the number of rate-limiting enzymes as inhibitor targets, All enzymes (Target) represent the number of all the inhibitor targets of metabolic enzymes, All RL enzymes represent the number of all rate-limiting enzymes, RL enzymes (Target) by RL enzymes (Initiator) represent the number of target rate-limiting enzymes which are inhibited by the products of other rate-limiting enzymes, RL enzymes (Target) by All enzymes (Initiator) represent the number of target rate-limiting enzymes which are inhibited by the products of all the metabolic enzymes, it is evident that from the first row, in total, only 18.7% of inhibitor initiators were rate-limiting enzymes. Based on the criterion of how many enzyme targets can be inhibited, the effectiveness of rate-limiting enzymes was tested in inhibitory networks. In total, the products of rate-limiting enzymes inhibited 75.34% of all inhibited targets from all the pathway groups in human liver. Further hypergeometric tests confirmed that the *in vivo *inhibitors were statistically enriched in the products of rate-limiting enzymes, relative to all the metabolites in human liver (all P-values < 0.001). On the other hand, although only a small proportion of targets were rate-limiting enzymes (Figure 3a), these targets of rate-limiting enzymes were more likely to be inhibited by the products of other rate-limiting enzymes.

From the aspect of cross-inhibition between pathways, we also found potential high efficiencies of rate-limiting enzymes as inhibitor initiators in inhibitory networks. Only one effectiveness ratio was lower than 60%, and all the remaining 35 ratios were greater than sixty percent (Figure 3b). The average ratio was 74.3%, which revealed that the rate-limiting enzymes, as inhibition providers, covered more than 74.3% of the cross-inhibition targets. For efficient metabolism, it is crucial for a cell to maintain a precise balance between different pathways. The high effectiveness of rate-limiting enzymes for cross-inhibition between pathways highlights the role of cross-pathway feedback regulation in maintaining the balance between different pathways.

## Discussion

In summary, we provide a basic pathway distribution for rate-limiting enzymes and branch points in human liver, and demonstrate the extensive topological links between rate-limiting enzymes and branch points. Over 76% of branch points are surrounded by rate-limiting enzymes. Several rate-limiting enzymes, such as isocitrate dehydrogenase, inosine 5'-monophosphate dehydrogenase and CDP-DAG synthase, are reported both to occur in branch points and to be regulated in these pathways [14-16,32,33]. As rate-limiting enzymes are often extensively regulated [34], such as by transcription factors and post-translational modifications, their influence on branch points may also be regulated in response to metabolic signals.

In addition, branch points show higher average degree, betweenness centrality and closeness centrality than those of all the metabolites in human liver. All these properties give branch points more power to influence the conversions among other compounds. Higher betweenness centrality means that the compounds have a higher probability of passing information between compound pairs in a metabolic network. Therefore, a compound in the shortest pathway between two given compounds is more likely to be recruited than compounds in longer pathways. Branch points as a whole are more likely to occur in such short conversion pathways. Since closeness measures the average number of steps needed to travel to other vertices, branch points are likely to receive information more quickly than other compounds in a diffusion process. The main reason why the averages of the three types of centrality from inhibitors are very high is because some inhibitors are located at branch points. Combining the influence of rate-limiting enzymes on branch points and the influence of branch points on other compounds, it seems that metabolic rate-limiting signals could impact the metabolic network in a hierarchal way.

Since rate-limiting enzymes often react at a relatively low velocity, it was assumed that the enzymes after branch points are often potentially regulatory [35]. However, our results showed no notable differences between the numbers of rate-limiting enzymes located before and after branch points in human liver. If we regard rate-limiting enzymes as potential regulatory targets, there would be no bias between the numbers of rate-limiting points directly before and after branch points, as we showed (Table 1). All the enzymes surrounding certain branch points influence the branch flux. It is logical that enzymes directly before branch points can control the production of branch points and thus influence the branch point concentration in a cell; conversely, it is also reasonable for enzymes after branch points to consume them and reduce their concentration in a cell.

Despite the capacity to influence metabolite flux via branch points, we also found that rate-limiting enzymes play important roles in enzyme inhibiting networks. The regulatory properties can be considered from two major aspects, regulability and regulatory capacity. The first describes how effectively the activity of the enzyme considered can be changed via other regulatory signals; the latter describes how effectively changes in the activity of the enzyme are transmitted to the rest of the system [18]. Since nearly half of the *in vivo *inhibitors are products of rate-limiting enzymes in human liver, these enzymes as a whole are easily able to initiate inhibitory regulation and transmit metabolic signals to other enzymes. Although only a small proportion of rate-limiting enzymes take part in inhibitory networks as inhibitor initiators, they cover over 75% of the *in vivo *inhibited targets. Further analysis of cross-inhibition between pathways confirmed the regulability and regulatory capacity of rate-limiting enzymes to balance the different metabolite fluxes from different pathways, which provide a metabolic basis to form a self-regulatory system. Since enzyme inhibition is a short-term form of regulation, which seldom involves any transcription or translation level events, it provides a mechanism to rapidly transmit metabolic signals and to balance the metabolites from different groups of pathway.

Further, it is interesting that rate-limiting enzymes as a whole are likely to be inhibited by their own products. This provides clues that the rate-limiting enzymes show some modularity in metabolic inhibitory networks. Since the products of these enzymes are always produced in a rate-limiting way, depending on the metabolic environment, their inhibitory effects may also be initiated by metabolic signals in a rate-limiting way.

From the view of inhibiting their targets, rate-limiting enzymes show high regulability and are easily reached by the inhibitors produced by other rate-limiting enzymes. Combining their regulability and regulatory capacity in compound conversion and inhibitory networks, rate-limiting enzymes are ideal regulatory molecules in the metabolic network. As we showed in the RLEdb, all 96 human rate-limiting enzymes were related to diseases; this may be a consequence of their central role in the control of metabolic flux and regulation.

## Conclusion

In conclusion, our systematic findings show that rate-limiting enzymes as a whole surround over three-quarters of the branch points in the metabolic network of human liver, therefore they can influence the flux through the branch points. Since nearly half of the *in vivo *enzyme inhibitors are produced by rate-limiting enzymes in human liver, thus these enzymes can initiate inhibitory regulation and then influence metabolic flux through these inhibitors.

## Methods

### Human liver expressing enzymes and compounds dataset

To get a reliable enzyme dataset, the entire list of genes expressed in liver was extracted from the HPRD database (23rd Feb 2007) [24]. Then 687 liver enzymes were collected after mapping all the genes to enzymes via KEGG ligand database 44.0 [26]. The 1033 natural products of these enzymes were extracted from KEGG ligand database 44.0.

### Manually curated branch points dataset

A branch point is defined to be any compound connected with three or more enzymes, at least one of which should be able to produce that compound and one that can consume it. However, many compounds such as ATP, reach the criteria easily; so, the 39 most common compounds were excluded: i) the 28 compounds which take part in more than 100 reactions; ii) the 4 too general compounds including RNA, DNA, Protein and Peptide; iii) the remaining 7 energy metabolism related nucleoside monophosphates, Nucleoside diphosphates and Nucleoside triphosphates (Additional file 1). Therefore, using this definition of branch points, 261 potential branch points were curated from the reference maps of the KEGG pathway. To get a branch point dataset for human liver based on 261 potential branch points, the tissue expression profiles of all the surrounding enzymes for each branch point were checked. If three or more metabolic enzymes in human liver occurred around a certain potential branch point, and these enzymes produced and consumed the compound, it was considered to be a branch point for human liver (Additional file 2).

### Collection of rate-limiting enzymes

The 147 rate-limiting enzymes from human were collected from rate-limiting enzymes database (RLEdb), which is the first literature-based rate-limiting enzyme database [19]. The 96 rate-limiting enzymes expressed in liver were isolated using the liver enzyme expression dataset (Additional file 3).

### Collection of *in vivo *enzyme inhibitors from the BRENDA database

The enzyme inhibitor information was extracted from BRENDA database 7.1 [20]. Organism-specific inhibitors were recorded in a given EC code in the BRENDA database. A similar semi-automatic method was used to convert free text inhibitor information to KEGG compound identifiers as described in previous studies [25,26]. For each enzyme, if the inhibitor description from BRNEDA exactly matched a KEGG compound name, we assigned the KEGG compound to that description. Then we grouped all assigned KEGG compounds together by their KEGG compound ID and checked all the mapping results manually. The same method was applied to the organism description from BRENDA.

However, many man-made inhibitors such as EDTA cannot be produced *in vivo*. We therefore selected the dataset of all human liver inhibitors by *in vivo *enzyme products in human liver. Although some inhibitors were enzyme products, they just inhibited other proteins, not metabolic enzymes. We also excluded such inhibitors from the final dataset as they did not have inhibiting effects in the human liver metabolic network. After collecting all the enzyme inhibitors, we isolated enzyme inhibiting pairs among which one was the inhibitor provider enzyme and the other was the inhibited target enzyme.

### Construction of the compound conversion network for human liver

We constructed a compound conversion network for human liver using compound pairs taken from the KEGG ligand database on 6th Nov 2007 [26]. This database is currently the only one available that records compound pair conversion directly. From the ligand database, we first got the rpair relations between compounds. The same procedure was executed by filtering out the 39 most common compounds and other compounds that are not the products of human liver according to the compound dataset of human liver.

### Network analysis with Pajek

We used the network analysis tool Pajek to calculate the normalized degree, betweenness centrality and closeness centrality of the compound conversion network in human liver [31,36].

### Statistical significance test

Throughout the paper, the hypergeometric test was used to calculate whether a given set of object pairs had a different frequency of annotation pairs than would be expected by chance, given the sample sizes involved and the expected frequency of such pairs. All p-values reported were calculated using the hypergeometric test for enrichment carried out using R package 2.6.2 [37]. A low p-value indicates that the association between annotation pairs is statistically significant.

The unequal t-tests were used to determine whether the difference in average mean values of two unequal variables x and y is statistically significant. The null hypothesis is that x and y are not different, and the p-value is the probability of getting a value of the test statistic as extreme as or more extreme than that observed by chance alone, if the null hypothesis is true. The statistical tests were performed using R package 2.6.2.

## Competing interests

The authors declare that they have no competing interests.

## Authors' contributions

MZ carried out all analyses and helped write the manuscript. HQ conceived of the analysis and helped write the manuscript.

## Note

Other papers from the meeting have been published as part of *BMC Bioinformatics* Volume 10 Supplement 15, 2009: Eighth International Conference on Bioinformatics (InCoB2009): Bioinformatics, available online at http://www.biomedcentral.com/1471-2105/10?issue=S15.

## Supplementary Material

Additional file 1 **Top 39 common compounds**. The following 39 common compounds were excluded from the compound conversion and metabolic inhibitory networks: i) 28 which take part in more than 100 reactions; ii) 4 which are too general, i.e. RNA, DNA, protein and peptide; and iii) the remaining 7 were energy metabolism-related nucleoside monophosphates, nucleoside diphosphates and nucleoside triphosphates. Click here for file

Additional file 2 **Branch points curated from KEGG pathways**. The 132 branch points in human liver are shown in Additional file 2. Click here for file

Additional file 3 **Rate-limiting enzymes from the literature**. The curated 96 rate-limiting enzymes from human liver are listed in Additional file 3. Click here for file

## References

[B1] He F Human liver proteome project: plan, progress, and perspectives Mol Cell Proteomics 2005 4 12 1841 1848 10.1074/mcp.R500013-MCP200 16118399

[B2] Fernie AR Geigenberger P Stitt M Flux an important, but neglected, component of functional genomics Curr Opin Plant Biol 2005 8 2 174 182 10.1016/j.pbi.2005.01.008 15752998

[B3] Fiehn O Metabolomics--the link between genotypes and phenotypes Plant Mol Biol 2002 48 1-2 155 171 10.1023/A:1013713905833 11860207

[B4] Almaas E Kovacs B Vicsek T Oltvai ZN Barabasi AL Global organization of metabolic fluxes in the bacterium Escherichia coli Nature 2004 427 6977 839 843 10.1038/nature02289 14985762

[B5] Sauer U High-throughput phenomics: experimental methods for mapping fluxomes Curr Opin Biotechnol 2004 15 1 58 63 10.1016/j.copbio.2003.11.001 15102468

[B6] Bro C Nielsen J Impact of 'ome' analyses on inverse metabolic engineering Metab Eng 2004 6 3 204 211 10.1016/j.ymben.2003.11.005 15256210

[B7] Krebs HA Kornberg HL Energy Transformations in Living Matter 1957 13609573

[B8] Th Bücher WR Gleichgewicht und Ungleichgewicht im System der Glykolyse Angewandte Chemie 1963 75 19 881 893 10.1002/ange.19630751902

[B9] Higgins JJ In control of energy metabolism 1965

[B10] Walsh K Koshland DE Jr Determination of flux through the branch point of two metabolic cycles. The tricarboxylic acid cycle and the glyoxylate shunt J Biol Chem 1984 259 15 9646 9654 6378912

[B11] Coleman J Raetz CR First committed step of lipid A biosynthesis in Escherichia coli: sequence of the lpxA gene J Bacteriol 1988 170 3 1268 1274 3277952 10.1128/jb.170.3.1268-1274.1988 PMC210902

[B12] Heijnen JJ van Gulik WM Shimizu H Stephanopoulos G Metabolic flux control analysis of branch points: an improved approach to obtain flux control coefficients from large perturbation data Metab Eng 2004 6 4 391 400 10.1016/j.ymben.2004.07.002 15491867

[B13] P Koll RBDB Regulation of metabolic branch points of aromatic amino acid biosynthesis in Pichia guilliermondii Journal of Basic Microbiology 1988 28 9-10 619 627 10.1002/jobm.3620280915 2907046

[B14] Barsotti C Pesi R Giannecchini M Ipata PL Evidence for the involvement of cytosolic 5'-nucleotidase (cN-II) in the synthesis of guanine nucleotides from xanthosine J Biol Chem 2005 280 14 13465 13469 10.1074/jbc.M413347200 15699053

[B15] Gu JJ Gathy K Santiago L Chen E Huang M Graves LM Mitchell BS Induction of apoptosis in IL-3-dependent hematopoietic cell lines by guanine nucleotide depletion Blood 2003 101 12 4958 4965 10.1182/blood-2002-08-2547 12609835

[B16] Stueland CS Gorden K LaPorte DC The isocitrate dehydrogenase phosphorylation cycle. Identification of the primary rate-limiting step J Biol Chem 1988 263 36 19475 19479 3058700

[B17] Van Dien S Schilling CH Bringing metabolomics data into the forefront of systems biology Mol Syst Biol 2006 2 10.1038/msb4100078 16788596 PMC1681510

[B18] Hofmeyr JH Cornish-Bowden A Quantitative assessment of regulation in metabolic systems Eur J Biochem 1991 200 1 223 236 10.1111/j.1432-1033.1991.tb21071.x 1879427

[B19] Zhao M Chen X Gao G Tao L Wei L RLEdb: a database of Rate-Limiting Enzymes and their regulation in Human, Rat, Mouse, Yeast and E. coli Cell Res 2009 19 6 793 795 10.1038/cr.2009.61 19468287

[B20] Chang A Scheer M Grote A Schomburg I Schomburg D BRENDA, AMENDA and FRENDA the enzyme information system: new content and tools in 2009 Nucleic Acids Res 2008 18984617 10.1093/nar/gkn820 PMC2686525

[B21] Masunari N Fujiwara S Nakata Y Furukawa K Kasagi F Effect of angiotensin converting enzyme inhibitor and benzodiazepine intake on bone loss in older Japanese Hiroshima J Med Sci 2008 57 1 17 25 18578363

[B22] Gantenbein MH Bauersfeld U Baenziger O Frey B Neuhaus T Sennhauser F Bernet V Side effects of angiotensin converting enzyme inhibitor (captopril) in newborns and young infants J Perinat Med 2008 18605972 10.1515/JPM.2008.064

[B23] Gutteridge A Kanehisa M Goto S Regulation of metabolic networks by small molecule metabolites BMC Bioinformatics 2007 8 1 88 10.1186/1471-2105-8-88 17352833 PMC1839110

[B24] Prasad TS Goel R Kandasamy K Keerthikumar S Kumar S Mathivanan S Telikicherla D Raju R Shafreen B Venugopal A Human Protein Reference Database--2009 update Nucleic Acids Res 2008 10.1093/nar/gkn892 PMC2686490 18988627

[B25] Kanehisa M Goto S Hattori M Aoki-Kinoshita KF Itoh M Kawashima S Katayama T Araki M Hirakawa M From genomics to chemical genomics: new developments in KEGG Nucleic Acids Res 2006 34 Database D354 357 10.1093/nar/gkj102 16381885 PMC1347464

[B26] Aoki KF Kanehisa M Using the KEGG database resource Curr Protoc Bioinformatics 2005 Chapter 1 Unit 1 12 18428742 10.1002/0471250953.bi0112s11

[B27] Koyuturk M Szpankowski W Grama A Assessing significance of connectivity and conservation in protein interaction networks J Comput Biol 2007 14 6 747 764 10.1089/cmb.2007.R014 17691892

[B28] Barabasi AL Albert R Emergence of scaling in random networks Science 1999 286 5439 509 512 10.1126/science.286.5439.509 10521342

[B29] Yook SH Oltvai ZN Barabasi AL Functional and topological characterization of protein interaction networks Proteomics 2004 4 4 928 942 10.1002/pmic.200300636 15048975

[B30] Barabasi AL Oltvai ZN Network biology: understanding the cell's functional organization Nat Rev Genet 2004 5 2 101 113 10.1038/nrg1272 14735121

[B31] W de Nooy AM Batagelj V Exploratory Social Network Analysis with Pajek 2005

[B32] Heacock AM Agranoff BW CDP-diacylglycerol synthase from mammalian tissues Biochim Biophys Acta 1997 1348 1-2 166 172 9370329 10.1016/s0005-2760(97)00096-9

[B33] Huang CY Christensen BM Chen CC Role of dopachrome conversion enzyme in the melanization of filarial worms in mosquitoes Insect Mol Biol 2005 14 6 675 682 10.1111/j.1365-2583.2005.00597.x 16313567

[B34] Newsholme EAaSC Regulation in Metabolism 1973

[B35] Heinrich R Rapoport SM Rapoport TA Metabolic regulation and mathematical models Prog Biophys Mol Biol 1977 32 1 1 82 343173

[B36] Chapter about Pajek V Batagelj AM Pajek - Analysis and Visualization of Large Networks Graph Drawing Software 2003 77 103

[B37] Team RDC R: A Language and Environment for Statistical Computing 2008

