# Supplementary material for: Human liver rate-limiting enzymes influence metabolic flux via branch points and inhibitors
Source: BMC Genomics. 2009 Dec 3;10(Suppl 3):S31. doi: 10.1186/1471-2164-10-S3-S31 (PMC2788385; doi:10.1186/1471-2164-10-S3-S31)
Supplement: Additional file 1 — Top 39 common compounds. The following 39 common compounds were excluded from the compound conversion and metabolic inhibitory networks: i) 28 which take part in more than 100 reactions; ii) 4 which are too general, i.e. RNA, DNA, protein and peptide; and iii) the remaining 7 were energy metabolism-related nucleoside monophosphates, nucleoside diphosphates and nucleoside triphosphates. [file 1471-2164-10-S3-S31-S1.pdf]

Additional 1 - Top 39 common compounds

The following 39 common compounds were excluded from the compound conversion and metabolic inhibitory networks: i) 28 which take part in more than 100 reactions; ii) 4 which are too general, i.e. RNA, DNA, protein and peptide; and iii) the remaining 7 were energy metabolism-related nucleoside monophosphates, nucleoside diphosphates and nucleoside triphosphates.

Compound_ID	Name	Reactions	Rank
C00001	H_2O	2418	1
C00080	H+	1386	2
C00007	O2	961	3
C00006	NADP+	791	4
C00005	NADPH	788	5
C00003	NAD+	700	6
C00004	NADH	691	7
C00002	ATP	479	8
C00011	CO2	448	9
C00010	Coenzyme A	415	10
C00009	Phosphate	407	11
C00015	UDP	361	12
C00008	ADP	340	13
C00014	NH3	306	14
C00013	Pyrophosphate	303	15
C00019	S-Adenosyl-L-methionine	265	16
C00021	S-Adenosyl-L-homocysteine	253	17
C00028	Acceptor	195	18
C00030	Reduced acceptor	193	19
C00027	H2O2	169	20
C00020	AMP	166	21
C00026	2-Oxoglutarate	162	22
C00022	Pyruvate	155	23
C00024	Acetyl-CoA	146	24
C00025	L-Glutamic acid	133	25
C00029	UDP-glucose	130	26
C00055	CMP	103	27
C00033	Acetate	101	28
C00035	GDP	82	31
C00063	CTP	31	69
C00044	GTP	31	70
C00105	UMP	28	74
C00075	UTP	25	86

Compound_ID	Name	Reactions	Rank
C00144	GMP	16	119
C00112	CDP	13	194
C00046	RNA	12	221
C00017	Protein	19	132
C00039	DNA	10	278
C00012	Peptide	7	490
