# Supplementary material for: Human liver rate-limiting enzymes influence metabolic flux via branch points and inhibitors
Source: BMC Genomics. 2009 Dec 3;10(Suppl 3):S31. doi: 10.1186/1471-2164-10-S3-S31 (PMC2788385; doi:10.1186/1471-2164-10-S3-S31)
Supplement: Additional file 2 — Branch points curated from KEGG pathways. The 132 branch points in human liver are shown in Additional file 2. [file 1471-2164-10-S3-S31-S2.pdf]

Additional 2 - 132 human liver branch points

Compound_ID from KEGG Lignad	Name	Fomular
C00037	Glycine	C2H5NO2
C00049	L-Aspartate	C4H7NO4
C00051	Glutathione	C10H17N3O6S
C00062	L-Arginine	C6H14N4O2
C00064	L-Glutamine	C5H10N2O3
C00065	L-Serine	C3H7NO3
C00073	L-Methionine	C5H11NO2S
C00077	L-Ornithine	C5H12N2O2
C00081	ITP	C10H15N4O14P3
C00083	Malonyl-CoA	C24H38N7O19P3S
C00084	Acetaldehyde	C2H4O
C00085	D-Fructose 6-phosphate	C6H13O9P
C00095	D-Fructose	C6H12O6
C00097	L-Cysteine	C3H7NO2S
C00099	beta-Alanine	C3H7NO2
C00100	Propanoyl-CoA	C24H40N7O17P3S
C00101	Tetrahydrofolate	C19H23N7O6
C00103	D-Glucose 1-phosphate	C6H13O9P
C00104	IDP	C10H14N4O11P2
C00111	Glycerone phosphate	C3H7O6P
C00116	Glycerol	C3H8O3
C00120	Biotin	C10H16N2O3S
C00124	D-Galactose	C6H12O6
C00129	Isopentenyl diphosphate	C5H12O7P2
C00130	IMP	C10H13N4O8P
C00131	dATP	C10H16N5O12P3
C00134	Putrescine	C4H12N2
C00135	L-Histidine	C6H9N3O2
C00136	Butanoyl-CoA	C25H42N7O17P3S
C00143	5,10-Methylenetetrahydrofolate	C20H23N7O6
C00148	L-Proline	C5H9NO2
C00153	Nicotinamide	C6H6N2O
C00154	Palmitoyl-CoA	C37H66N7O17P3S
C00157	Phosphatidylcholine	C10H18NO8PR2
C00162	Fatty acid	CHO2R
C00166	Phenylpyruvate	C9H8O3
C00187	Cholesterol	C27H46O
C00195	N-Acylsphingosine	C19H36NO3R

Compound_ID from KEGG Lignad	Name	Fomular
C00206	dADP	C10H15N5O9P2
C00212	Adenosine	C10H13N5O4
C00219	(5Z,8Z,11Z,14Z)-Icosatetraenoic acid	C20H32O2
C00222	3-Oxopropanoate	C3H4O3
C00229	Acyl-carrier protein	HSR
C00234	10-Formyltetrahydrofolate	C20H23N7O7
C00236	3-Phospho-D-glyceroyl phosphate	C3H8O10P2
C00239	dCMP	C9H14N3O7P
C00250	Pyridoxal	C8H9NO3
C00256	(R)-Lactate	C3H6O3
C00262	Hypoxanthine	C5H4N4O
C00267	alpha-D-Glucose	C6H12O6
C00275	D-Mannose 6-phosphate	C6H13O9P
C00280	Androst-4-ene-3,17-dione	C19H26O2
C00286	dGTP	C10H16N5O13P3
C00299	Uridine	C9H12N2O6
C00319	Sphingosine	C18H37NO2
C00330	Deoxyguanosine	C10H13N5O4
C00334	4-Aminobutanoate	C4H9NO2
C00341	Geranyl diphosphate	C10H20O7P2
C00361	dGDP	C10H15N5O10P2
C00362	dGMP	C10H14N5O7P
C00363	dTDP	C10H16N2O11P2
C00364	dTMP	C10H15N2O8P
C00365	dUMP	C9H13N2O8P
C00385	Xanthine	C5H4N4O2
C00410	Progesterone	C21H30O2
C00416	Phosphatidate	C5H7O8PR2
C00427	Prostaglandin H2	C20H32O5
C00445	5,10-Methenyltetrahydrofolate	C20H22N7O6
C00448	trans,trans-Farnesyl diphosphate	C15H28O7P2
C00454	NDP	C5H11O10P2R
C00455	Nicotinamide D-ribonucleotide	C11H15N2O8P
C00468	Estrone	C18H22O2
C00475	Cytidine	C9H13N3O5
C00535	Testosterone	C19H28O2
C00555	4-Aminobutanal	C4H9NO
C00577	D-Glyceraldehyde	C3H6O3
C00601	Phenylacetaldehyde	C8H8O
C00627	Pyridoxine phosphate	C8H12NO6P

**Compound_ID from KEGG Lignad**	**Name**	**Fomular**
C00632	3-Hydroxyanthranilate	C7H7NO3
C00637	Indole-3-acetaldehyde	C10H9NO
C00641	1,2-Diacyl-sn-glycerol	C5H6O5R2
C00645	N-Acetyl-D-mannosamine	C8H15NO6
C00647	Pyridoxamine phosphate	C8H13N2O5P
C00655	Xanthosine 5'-phosphate	C10H13N4O9P
C00668	alpha-D-Glucose 6-phosphate	C6H13O9P
C00705	dCDP	C9H15N3O10P2
C00718	Amylose	(C6H10O5)n
C00721	Dextrin	(C12H20O10)n
C00735	Cortisol	C21H30O5
C00836	Sphinganine	C18H39NO2
C00877	Crotonoyl-CoA	C25H40N7O17P3S
C00881	Deoxycytidine	C9H13N3O4
C00942	3',5'-Cyclic GMP	C10H12N5O7P
C01120	Sphinganine 1-phosphate	C18H40NO5P
C01157	trans-4-Hydroxy-L-proline	C5H9NO3
C01181	4-Trimethylammoniobutanoate	C7H16NO2
C01185	Nicotinate D-ribonucleotide	C11H15NO9P
C01209	Malonyl-[acyl-carrier protein]	C3H3O3SR
C01227	3beta-Hydroxyandrost-5-en-17-one	C19H28O2
C01243	1D-myo-Inositol 1,3,4-trisphosphate	C6H15O15P3
C01245	D-myo-Inositol 1,4,5-trisphosphate	C6H15O15P3
C01272	1D-myo-Inositol 1,3,4,5-tetrakisphosphate	C6H16O18P4
C01277	1-Phosphatidyl-1D-myo-inositol 4-phosphate	C11H18O16P2R2
C01346	dUDP	C9H14N2O11P2
C01794	Choloyl-CoA	C45H74N7O20P3S
C01885	1-Acylglycerol	C4H7O4R
C02686	Galactosylceramide	C25H46NO8R
C03069	3-Methylcrotonyl-CoA	C26H42N7O17P3S
C03227	3-Hydroxy-L-kynurenine	C10H12N2O4
C03460	2-Methylprop-2-enoyl-CoA	C25H40N7O17P3S
C03939	Acetyl-[acyl-carrier protein]	
C04230	1-Acyl-sn-glycero-3-phosphocholine	C9H20NO7PR
C04637	1-Phosphatidyl-D-myo-inositol 4,5-bisphosphate	C11H19O19P3R2
C05345	beta-D-Fructose 6-phosphate	C6H13O9P
C05378	beta-D-Fructose 1,6-bisphosphate	C6H14O12P2
C05451	7alpha-Hydroxy-5beta-cholestan-3-one	C27H46O2
C05453	7alpha,12alpha-Dihydroxy-5beta-cholestan-3-one	C27H46O3
C05457	7alpha,12alpha-Dihydroxycholest-4-en-3-one	C27H44O3

Compound_ID from KEGG Lignad	Name	Fomular
C05473	11beta,21-Dihydroxy-3,20-oxo-5beta-pregnan-18-al	C21H30O5
C05479	5beta-Pregnane-3,20-dione	C21H32O2
C05634	5-Hydroxyindoleacetaldehyde	C10H9NO2
C05947	L-erythro-4-Hydroxyglutamate	C5H9NO5
C06002	(S)-Methylmalonate semialdehyde	C4H6O3
C06124	Sphingosine 1-phosphate	C18H38NO5P
C06205	1,2-Dihydronaphthalene-1,2-diol	C10H10O2
C07490	Trichloroethanol	C2H3Cl3O
C14786	(1R,2S)-Naphthalene 1,2-oxide	C10H8O
C14787	(1S,2R)-Naphthalene 1,2-oxide	C10H8O
C14800	1-Nitronaphthalene-5,6-oxide	C10H7NO3
C14839	Bromobenzene-3,4-oxide	C6H5BrO
C14840	Bromobenzene-2,3-oxide	C6H5BrO
C14852	Benzo[a]pyrene-7,8-diol	C20H14O2
