# Supplementary material for: Human liver rate-limiting enzymes influence metabolic flux via branch points and inhibitors
Source: BMC Genomics. 2009 Dec 3;10(Suppl 3):S31. doi: 10.1186/1471-2164-10-S3-S31 (PMC2788385; doi:10.1186/1471-2164-10-S3-S31)
Supplement: Additional file 3 — Rate-limiting enzymes from the literature. The curated 96 rate-limiting enzymes from human liver are listed in Additional file 3. [file 1471-2164-10-S3-S31-S3.pdf]

Additional 3 - 96 human liver rate-limiting enzymes

Note: The RLEdb address is HTTP://rle.cbi.pku.edu.cn

The content in the fourth column is fomated as tissue[HPRD_gene_ID]#

EC code	RLEdb ID	Name	Tissue exression from HPRD
1.1.1.1	RL00087	alcohol dehydrogenase	Colon[00064]#Uterus[00064]#Gastrointestinal Tract[00068]#Fetus[00064]#Skin[00066]#Ileum[00064]#Testis[00064]#Liver[00064,00063,00066,00068,00065,00067]#Placenta[00064]#Brain[00064]#Stomach[02515,00064,00067]#
1.1.1.105	RL00161	cis retinol dehydrogenase	Thymus[03370]#Colon[03370]#Nervous system[03370]#Uterus[09431,03370]#Intestine[03370]#Testis[09431,03370]#Liver[09431,03370]#Brain[09431]#Prostate[09431]#Spleen[09431,03370]#Kidney[03370]#Mammary gland[09431,03370]#Adrenal gland[09431]#Heart[03370]#Lung[09431]#Placenta[09431]#Retina[03370]#
1.1.1.145	RL00069	hydroxy delta steroid dehydrogenase	Uterus[01941]#Intestine[01941]#Testis[01941]#Liver[01941]#Muscle[01941]#Brain[01941]#Prostate[01941,04920]#Epididymis[01941]#Pulmonary artery[04920]#Adipose tissue[01941]#Kidney[01941]#Adrenal gland[01941]#Mammary gland[01941,04920]#Heart[01941]#Lung[01941]#Ovary[01941]#Skin[01941,04920]#Placenta[01941,04920]#Vascular endothelium[04920]#Sebaceous gland[04920]#Pituitary gland[01941]#
1.1.1.205	RL00257	Inosine monophosphate dehydrogenase	Thymus[00895]#Colon[00895]#Fetus[00895]#Leukocyte[08853,00895]#Testis[00895]#Skeletal muscle[00895]#Liver[00895]#Brain[08853,00895]#Spleen[08853,00895]#Prostate[00895]#Small intestine[00895]#Kidney[00895]#T Cell[00895]#Lung[00895]#Heart[00895]#Ovary[00895]#Pancreas[00895]#Placenta[00895]#Lymphocyte[08853]#
1.1.1.21	RL00250	aldose reductase/NADPH-dependent aldo-keto reductase	Thymus[00071]#Colon[00071]#Lens[00071]#Schwann cell[00071]#Cerebral cortex[00071]#Fetus[00071]#Aorta[00071]#Leukocyte[00071]#Testis[00071]#Skeletal muscle[00071]#Liver[00071]#Renal tubule[00071]#Red blood cell[00071]#Retinal pigmented epithelial cell[00071]#Muscle[00071]#Brain[00071]#Spleen[00071]#Prostate[00071]#Small intestine[00071]#Kidney[00071]#Smooth muscle[00071]#Sympathetic nervous system[00071]#Heart[00071]#Lung[00071]#Ovary[00071]#Placenta[00071]#Retina[00071]#Midbrain[00071]#Basal nuclei[00071]#
1.1.1.22	RL00128	uridine diphosphoglucose dehydrogenase	Thymus[04535]#Colon[04535]#Leukocyte[04535]#Testis[04535]#Skeletal muscle[04535]#Liver[04535]#Brain[04535]#Spleen[04535]#Prostate[04535]#Small intestine[04535]#Kidney[04535]#Lung[04535]#Heart[04535]#Pancreas[04535]#Ovary[04535]#Placenta[04535]#

EC code	RLEdb ID	Name	Tissue exression from HPRD
1.1.1.34	RL00230	3-hydroxy-methylglutaryl coenzyme A	Monocyte[00836]#Fetus[00836]#Skin fibroblast[00836]#Granulocyte[00836]#Leukocyte[00836]#Plasma[00836]#Ileum[00836]#Macrophage[00836]#Liver[00836]#Placenta[00836]#Adrenal cortex[00836]#Lung fibroblast[00836]#Umbilical vein endothelial cell[00836]#Lymphocyte[00836]#
1.1.1.35	RL00006	acyl-CoA dehydrogenase	Colon[08372,03514]#Occipital pole[02223]#Hippocampus[02223]#Corpus callosum[02223]#Cardiac muscle[08372,02223]#Fetus[06125]#Islets of Langerhans[08372]#Testis[08372,03514,02223]#Brain[08372,03514,02223]#Spleen[08372,03514]#Thalamus[02223]#Heart[08372,03514,02223]#Amygdala[02223]#Cerebellum[02223]#Lymphocyte[03514]#Pituitary gland[03514]#Thymus[08372,03514]#Uterus[03514]#Substantia nigra[02223]#Cerebral cortex[02223]#Bone cell[03514]#Spinal cord[02223]#Leukocyte[08372]#Caudate nucleus[02223]#Putamen[02223]#Intestine[03514]#Temporal lobe[02223]#Liver[08372,03514,02223]#Skeletal muscle[08372,03514,02223]#Prostate[08372,03514]#Kidney[08372,03514,02223,06125]#Lung[08372,03514,02223]#Pancreas[08372,03514,02223]#Ovary[08372,03514,02223]#Placenta[08372,03514,02223]#Subthalamic nucleus[02223]#Frontal lobe[02223]#
1.1.1.49	RL00271	glucose-6-phosphate dehydrogenase	Hematopoietic stem cell[02377]#Liver[02377]#Placenta[02377]#Red blood cell[02377]#Blood[02377]#
1.1.99.5	RL00066	glycerol phosphate dehydrogenase	Heart[00715]#Pancreas[00715]#Liver[00715]#Placenta[00715]#Muscle[00715]#
1.13.11.11	RL00279	tryptophan 2,3-dioxygenase	Liver[01847]#
1.14.13.17	RL00229	cholesterol 7alpha-hydroxylase	Kidney[00324]#Heart[00324]#Lung[00324]#Pancreas[00324]#Liver[00324]#Skeletal muscle[00324]#Placenta[00324]#Brain[00324]#
1.14.13.39	RL00249	nitric oxide synthase	Nervous system[01224]#Lung epithelium[01225]#Uterus[01225]#Eye[01224]#Leukocyte[01225]#Hepatocyte[01225]#Skeletal muscle[01225]#Liver[01224]#Muscle[01226]#Brain[01226,01225,01224]#B Cell[01225]#Uterine myometrium[01225]#Platelet[01224]#Kidney[01226]#Blood vessel[01224]#Airway epithelium[01225]#Smooth muscle[01225]#Monocyte[01225]#Endometrium[01225]#Neutrophil[01225,01224]#Synovial fluid[01225]#Lung[01224]#Heart[01226,01224]#Chondrocyte[01225]#Dental pulp[01225]#Melanocyte[01225]#Placenta[01225,01224]#Cerebellum[01225]#Retina[01225]#Gingival epithelium[01225]#
1.14.13.9	RL00009	Kynurenine hydroxylase	Kidney[04642]#Heart[04642]#Lung[04642]#Pancreas[04642]#Liver[04642]#Skeletal muscle[04642]#Placenta[04642]#Brain[04642]#
1.14.13.99	RL00221	CYP39A1	Liver[07067]#

EC code	RLEdb ID	Name	Tissue exression from HPRD
1.14.14.1	RL00227	Aromatase	Hippocampus[00477,00488,07101]#Jejunum[03159,05617]#Alpha cell[03159]#Aorta[00477]#Islets of Langerhans[03159]#Urinary bladder[00491]#Macrophage[03159,05617,00491]#Hepatocyte[00148]#Spleen[05942,03464]#Vascular system[00488]#Endometrium[05630,00491]#Nasal mucosa[00486,07454]#Vascular endothelium[03159]#Thymus[05942,03464]#Bronchial epithelial cell[11813,00484]#Syncytiotrophoblast[00488]#Occipital lobe[00477]#Substantia nigra[00477]#Leukocyte[05942,03464]#Intestine[05630,03464]#Caudate nucleus[00477]#Putamen[00477]#Liver[07453,07101,00487,00486,07454,03464,00477,00488,03085,05617,08518,03083,00490,07102,05942,03159,11813,00489,00484,03084,05630]#Blood[05617]#Muscle[03464]#Prostate[08518,00488,05942,05617,07454,03464,00491]#Astrocyte[00477]#Smooth muscle[03159]#Adrenal gland[03083]#Delta cell[03159]#Gastric mucosa[00489]#Lung[00487,07454,03464,00148,00477,03085,05617,03083,00490,05942,03159,11813,00489,00484,00491,05630]#Skin[05617]#Epidermis[08518,03085]#Ovary[03083,00488,03464,00148]#Cervix[05630]#Globus pallidus[00477]#Hair follicle[08518]#Midbrain[03083]#Atrium[00477]#Renal cortex[08518]#
1.14.19.1	RL00262	Stearoyl-CoA desaturase	Spinal cord[04941]#Fetus[09756,04941]#Skeletal muscle[09756,04941]#Liver[09756,04941]#Retinal pigmented epithelial cell[04941]#Brain[09756,04941]#Keratinocyte[04941]#Kidney[09756,04941]#Lung[09756,04941]#Heart[09756,04941]#Hair[04941]#Pancreas[09756,04941]#Skin[04941]#Trachea[04941]#Cerebellum[04941]#Placenta[09756,04941]#Retina[04941]#Sebaceous gland[04941]#
1.14.99.1	RL00228	cyclooxygenase	Colon[07518]#Fallopian tube[02599]#Occipital pole[07518]#Fetus[02599]#Testis[07518]#Brain[07518]#Small intestine[07518]#Uterine myometrium[02599]#Endometrium[02599]#Osteoblast[02599]#Heart[07518]#Cerebellum[07518]#Umbilical vein endothelial cell[02599]#Podocyte[02599]#Thymus[02599,07518]#Uterus[07518]#Cerebral cortex[07518]#Spinal cord[07518]#Skin fibroblast[02599]#Putamen[07518]#Temporal lobe[07518]#Liver[07518]#Skeletal muscle[07518]#Prostate[07518]#Kidney[07518]#Mammary gland[07518]#Monocyte[02599]#Lung[02599,07518]#Medulla oblongata[07518]#Pancreas[07518]#Placenta[07518]#Frontal lobe[07518]#Stomach[07518]#
1.14.99.7	RL00131	squalene epoxidase	Liver[11802]#

EC code	RLEdb ID	Name	Tissue exression from HPRD
1.17.1.4	RL00171	xanthine oxidoreductase	Small intestine[06363]#Plasma cell[06363]#Blood vessel[06363]#Mammary gland[06363]#Trophoblast[06363]#Heart[06363]#Liver[06363]#Skeletal muscle[06363]#Milk[06363]#Brain[06363]#
1.17.3.2	RL00172	xanthine oxidase	Small intestine[06363]#Plasma cell[06363]#Blood vessel[06363]#Mammary gland[06363]#Trophoblast[06363]#Heart[06363]#Liver[06363]#Skeletal muscle[06363]#Milk[06363]#Brain[06363]#
1.17.4.1	RL00267	ribonucleotide reductase	Small intestine[01587]#Colon[01587]#Kidney[01587]#Heart[01587]#Leukocyte[01587]#Lung[01587]#Testis[01587]#Pancreas[01587]#Ovary[01587]#Skeletal muscle[01587]#Liver[01587]#Placenta[01587]#Lymphocyte[01588]#Brain[01587]#Mammary epithelium[01587]#
1.2.1.3	RL00062	aldehyde dehydrogenase	Saliva[00004]#Salivary gland[02713]#Fetus[00005,00124]#Oesophagus[00004]#Hepatocyte[02713]#Testis[02713,00005]#Skeletal muscle[02713,07188]#Liver[02713,00005,00003,07188,00004,04109]#Keratinocyte[07188]#Brain[02713,00005,00003,07188,04109]#Muscle[00005,00003,04109]#Spleen[00003]#Kidney[02713,00005,00003,07188,00004,04109]#Parotid gland[02713]#Mammary gland[02713]#Adrenal gland[02713,04109]#Lung[02713,00003,07188,00004]#Heart[00005,00003,07188,04109]#Epidermis[07188]#Pancreas[02713,07188,04109]#Ovary[00003]#Hair[00004]#Placenta[02713,00005]#Stomach[02713,00003,00004]#
1.2.4.1	RL00180	pyruvate dehydrogenase	Kidney[02420]#Heart[01530,02420]#Skin fibroblast[01530,02420]#Testis[01531]#Skin[02420]#Liver[01530,02420]#Skeletal muscle[02420]#Brain[02420]#
1.3.1.2	RL00233	dihydropyrimidine dehydrogenase	Leukocyte[02036]#Intestine[02036]#Pancreas[02036]#Liver[02036]#
1.3.3.6	RL00222	acyl CoA oxidase	Skin fibroblast[02030]#Lung[02030]#Pancreas[02030]#Skeletal muscle[02030]#Liver[04552,02030]#Kidney[02030]#
1.3.99.13	RL00074	acyl coA dehydrogenase	Liver[01939]#
1.3.99.2	RL00073	medium chain acyl coA dehydrogenase	Liver[06053]#Kidney[06053]#
1.4.3.5	RL00012	pyridoxamine 5'-phosphate oxidase	Liver[04476]#
1.5.1.20	RL00187	methylenetetrahydrofolate reductase	Colon[06158]#Kidney[06158]#Heart[06158]#Intestine[06158]#Lung[06158]#Testis[06158]#Liver[06158]#Placenta[06158]#Brain[06158]#Muscle[06158]#Stomach[06158]#Spleen[06158]#

EC code	RLEdb ID	Name	Tissue exression from HPRD
1.9.3.1	RL00159	cytochrome c oxidase	Ubiquitous[03644]#Cardiac muscle[00481]#Fetus[12143,00466,02471,00467,04799]#Intestine[02471]#Testis[13088,12143,13090]#Liver[13088,02471,04799,00493,04812,00482,12143,00466,00467,00468]#Skeletal muscle[13088,02471,02469,00481,00493,00482,12143,00466,00467,04800,00468,03598]#Brain[12143,00466,00467,04799,00468,00494,00482]#Muscle[00466,04799]#Prostate[02469,00494]#Diaphragm[04812]#Kidney[12143,00466,02469,04812,00482]#Smooth muscle[00482]#Lung[12143,00466]#Heart[13088,02471,02469,00481,00493,04812,00482,12143,00466,04800,00468,03598]#Pancreas[12143,00466]#Placenta[12143]#
2.1.4.1	RL00164	arginine glycine transamidinase	Leukocyte[03838]#Pancreas[03838]#Liver[03838]#Kidney[03838]#
2.2.1.1	RL00013	transketolase	Kidney[02080]#Fetus[02080]#Leukocyte[06001]#Cornea[06001]#Heart[02080]#Lung[02080]#Islets of Langerhans[06001]#Pancreas[02080]#Skeletal muscle[02080]#Liver[02080,06001]#Placenta[02080]#Red blood cell[06001]#Brain[02080,06001]#
2.3.1.12	RL00002	pyruvate dehydrogenase complex	Kidney[02420]#Heart[01530,10578,02420]#Skin fibroblast[01530,02420]#Testis[01531]#Skin[02420]#Liver[01530,10578,02420]#Skeletal muscle[02420]#Brain[02006,02420]# Testis. Expressed in postmeiotic spermatogenic cells[P29803]#Ubiquitous[P08559]#
2.3.1.21	RL00236	carnitine palmitoyltransferase 1a	Small intestine[12315]#Colon[12315]#Ubiquitous[02802]#Heart[09065]#Testis[12315]#Ovary[12315]#Liver[02755]#Skeletal muscle[09065]#Brain[12315]#
2.3.1.26	RL00067	acyl coA : cholesterol acyltransferase	Duodenum[03202]#Bronchial epithelial cell[00033]#Thyroid follicular cell[00033]#Ubiquitous[00033]#Cerebral cortex[00033]#Spinal cord[00033]#Fetus[03202]#Testis[00033]#Macrophage[00033]#Hepatocyte[00033]#Liver[03202]#Cardiomyocyte[00033]#Renal tubule[00033]#Granulosa lutein cell[00033]#Small intestine[03202]#Villus[03202]#Enterocyte[03202]#Alveolus[00033]#Smooth muscle[00033]#Bile duct[00033]#Heart[00033]#Ileum[03202]#Urinary tract[00033]#Adrenal cortex[00033]#Pancreatic acinus[00033]#Pituitary gland[00033]#Stomach[00033]#
2.3.1.37	RL00237	5-aminolevulinate synthase	Hematopoietic stem cell[02356]#Bone marrow[02356]#Liver[00505]#Proerythroblast[00505]#Brain[00505]#
2.3.1.5	RL00244	N-acetyltransferase	Colon[02000]#Nervous system[00149]#Uterus[02000]#Mammary gland[02000]#Oesophagus[02000]#Intestine[02000,00149]#Lung[02000]#Liver[02000,00149]#Blood[00149]#Lymphocyte[00149]#Stomach[02000]#

EC code	RLEdb ID	Name	Tissue exression from HPRD
2.3.1.85	RL00094	fatty acid synthase	Colon[02567]#Duodenum epithelium[02567]#Zona fasciculata[02567]#Large intestine[02567]#Fetus[02567]#Vermiform appendix[02567]#Urinary bladder[02567]#Hepatocyte[02567]#Liver[02567]#Urothelium[02567]#Decidua[02567]#Adenohypophysis[02567]#Brain[02567]#Prostate[02567]#Epididymis[02567]#Cerebral neuron[02567]#Lactiferous duct[02567]#Astrocyte[02567]#Mammary gland[02567]#Seminiferous tubule[02567]#Endometrium[02567]#Lung[02567]#Mast cell[02567]#Cerebellum[02567]#Adrenal cortex[02567]#Adipocyte[02567]#Stomach[02567]#Sebaceous gland[02567]#
2.3.2.2	RL00102	gamma glutamyl transferase	Serum[01980]#Kidney[01980]#Monocyte[01980]#Ubiquitous[01980]#Fetus[01980]#Lung[01980]#Granulocyte[01980]#Plasma[01980]#Pancreas[01980]#Liver[01980]#Placenta[01980,00666]#Bile fluid[01980]#Brain[01980]#
2.3.3.10	RL00047	HMG CoA synthase	Heart[02580]#Colon[02580]#Testis[02580]#Liver[02580]#Kidney[02580]#Muscle[02580]#Fetus[07515]#
2.4.1.1	RL00004	glycogen phosphorylase	Serum[01986]#Heart[01986]#Liver[00720,01987]#Retina[00720]#Muscle[00720,01986]#Brain[00720,01986]#Fetus[00720]#
2.4.1.11	RL00243	glycogen synthase	Liver[00722]#Skeletal muscle[00721]#Endometrium[00721]#
2.4.1.17	RL00192	bilirubin uridine diphosphate glucuronosyltransferase	Biliary apparatus[08402,07566,07336]#Colon[08402,07566,07336,07072]#Jejunum[08933,07566,07336,07072]#Larynx[09397]#Fetus[05279]#Oesophagus epithelium[07193,02508]#Macrophage[07073]#Hepatocyte[02508]#Testis[07073,05931,07192,03545,02507]#Tonsil[07336,09397]#Tongue[07336,09397]#Brain[05279]#Spleen[05931]#Small intestine[07073,08933,07566,05931,07336,07192,07072]#Adipose tissue[07192,04345,02507]#Cerebellum[02508]#Duodenum[08933,07566,02508,07336,02507]#Uterus[07192,03545]#Large intestine[07336]#Intestine[08402]#Oesophagus[07336,07192,09397]#Liver[08402,08933,05931,07336,07192,04345,03545,02507,07193,07071,07073,07566,02508]#Olfactory system[05279]#Skeletal muscle[07073,05931]#Prostate[07073,05931,07192,04345,03545,02507]#Kidney[05931,07192,04345,03545,02507,07071,07073,02508]#Mammary gland[07073,08933,05931,07192,04345,03545,02507]#Adrenal gland[04345,03545,02507]#Bile duct[08933]#Oral mucosa[09397]#Lung[05931,02508,07192,04345,03545,09397,02507]#Pancreas[02508]#Skin[07073,07071,07192,04345,02507]#Ileum[07193,08933,07566,07336,07072]#Ovary[07073]#Placenta[05931,07192,03545,02507]#Stomach[07073,07566,05931,07336,09397]#

EC code	RLEdb ID	Name	Tissue exression from HPRD
2.4.1.22	RL00195	galactosyltransferase	Thymus[04928]#Colon[04928]#Uterus[04928]#Spinal cord[04928]#Salivary gland[04928]#Fetus[04928]#Leukocyte[04928]#Intestine[04928]#Testis[04928]#Skeletal muscle[04928]#Liver[04928,00659]#Muscle[04928]#Brain[04928]#Spleen[04928]#Prostate[04928]#Kidney[04928]#Mammary gland[04928,01024]#Adrenal gland[04928]#Heart[04928]#Lung[04928]#Ovary[04928]#Pancreas[04928]#Trachea[04928]#Milk[01024]#Placenta[04928,00659]#Thyroid gland[04928]#Stomach[04928]#
2.4.2.4	RL00282	Thymidine phosphorylase	Platelet[08833]#Liver[08833]#Placenta[08833]#Keratinocyte[08833]#Endometrium[08833]#
2.5.1.18	RL00049	glutathione s transferase	Colon[10401,05192,13613,00705,02700,02701,06899]#Lymph node[05192]#Ubiquitous[06899]#Cardiac muscle[00707]#Salivary gland[13613,00705]#Fetus[05192,13613,00705,00707,03436,04785,06899]#Aorta[00705,00707]#Hepatocyte[02700]#Urinary bladder[00705]#Testis[10401,13613,00705,00712,03436,13614,07290,00706,05192,00707,00709,00708,00711]#Islets of Langerhans[00614]#Myoblast[00710]#Red blood cell[00614]#Brain[10401,13613,00705,00712,03436,04785,05192,00706,00707,00709,00711,06899]#Spleen[10401,05192,13613,00705,03436]#Small intestine[10401,05192,13613,00705]#Heart[10401,13613,00705,00614,00712,04785,00706,05192,00709,06899]#Melanocyte[04785]#Adrenal medulla[05192]#Umbilical vein endothelial cell[03436]#Thyroid gland[05192,00705]#Pituitary gland[00705,00709]#Bronchial epithelial cell[00712]#Thymus[10401,05192,13613,00705]#Substantia nigra[00710]#Uterus[10401,00706,13613,00705]#Cerebral cortex[00706]#Spinal cord[13613]#Large intestine[06899]#Oesophagus[10401,00614]#Leukocyte[10401,05192,13613,00705,03436]#Vermiform appendix[05192]#Bone marrow[05192,13613,03436]#Liver[10401,13613,00705,00614,00712,0
2.5.1.6	RL00071	methionine adenosyl transferase	Liver[02013]#Lymphocyte[05701]#Ubiquitous[03275]#
2.5.1.61	RL00022	uroporphyrinogen synthase	Liver[01440]#Red blood cell[01440]#Spleen[01440]#
2.6.1.5	RL00174	tyrosine aminotransferase	Liver[11776]#Fetus[11776]#
2.7.1.1	RL00010	hexokinase	Kidney[00809]#Spermatozoa[00809]#Granulocyte[00806]#Lung[00806]#Testis[00809]#Skeletal muscle[03080]#Liver[00809,00806]#Placenta[00809]#Red blood cell[00809,00806]#Lymphocyte[00806]#Reticulocyte[00809]#Spleen[00806]#

EC code	RLEdb ID	Name	Tissue exression from HPRD
2.7.1.11	RL00238	phosphofructokinase	Thymus[01384]#Colon[01384]#Intestine[01384]#Leukocyte[01384,01385]#Testis[01384]#Liver[01384,01385]#Red blood cell[01385,01988]#Blood[01384]#Muscle[01988]#Brain[01384,01385]#Prostate[01384]#Platelet[01384,01385]#Kidney[01384,01385]#Heart[01384]#Pancreas[01384]#Ovary[01384]#Placenta[01384]#
2.7.1.2	RL00151	Glucokinase	Caudate nucleus[00680]#Beta cell[00680]#Liver[00680]#Hypothalamus[00680]#Cerebral cortex[00680]#Brain[00680]#
2.7.1.20	RL00213	adenosine kinase	Kidney[00039]#Leukocyte[00039]#Heart[00039]#Lung[00039]#Pancreas[00039]#Liver[00039]#Skeletal muscle[00039]#Placenta[00039]#Muscle[00039]#Brain[00039]#Spleen[00039]#
2.7.1.33	RL00185	pantothenate kinase	Kidney[07312,07549,05857,07313]#Ubiquitous[05857]#Heart[07312,07549,05857,07313]#Lung[05857,07313]#Testis[07549]#Pancreas[07312,05857,07313]#Skeletal muscle[07549]#Liver[07312,07549,05857,07313]#Placenta[07312,05857,07313]#Retina[05857]#Brain[07312,07549,05857,07313]#Muscle[07312,05857,07313]#
2.7.1.40	RL00136	pyruvate kinase	Small intestine[01529]#Serum[01529]#Colon[01529]#Adipose tissue[01529]#Kidney[01529]#Leukocyte[01529]#Lung[01529]#Ovary[11841]#Testis[01529]#Plasma[01529]#Islets of Langerhans[01529]#Liver[11841]#Red blood cell[01529]#Lymphocyte[01529]#Brain[01529]#Muscle[01529]#
2.7.1.48	RL00272	Uridine-cytidine nucleoside kinase	Small intestine[18256]#Kidney[01882,18256,15607]#Ubiquitous[15607]#Heart[01882,18256,15607]#Lung[01882,15607]#Macrophage[01882]#Pancreas[01882,15607]#Skeletal muscle[01882,18256,15607]#Liver[01882,18256,15607]#Placenta[01882,18256,15607]#Blood[01882]#Brain[01882,18256,15607]#Spleen[18256]#
2.7.1.60	RL00001	Bifunctional UDP-N-acetylglucosamine 2-epimerase/N-acetylmannosamine kinase (UDP-GlcNAc-2-epimerase/ManAc kinase)	Kidney[04825]#Heart[04825]#Lung[04825]#Pancreas[04825]#Liver[04825]#Placenta[04825]#Brain[04825]#Muscle[04825]#

EC code	RLEdb ID	Name	Tissue exression from HPRD
2.7.1.68	RL00070	phosphatidylinositol phosphate kinase	Nervous system[05834]#Hippocampus[17852]#Substantia nigra[17852]#Corpus callosum[17852]#Spinal cord[17852]#Fetus[17852,04470]#Caudate nucleus[17852]#Testis[17852]#Skeletal muscle[11931,17852,04470,04121]#Liver[11931,17852,05834,04470,04463]#Lung fibroblast[04463]#Muscle[04463]#Brain[11931,17852,05834,04470,04121,04463]#Spleen[05834]#Platelet[17852,04470]#Kidney[11931,17852,05834,04470,04121,04463]#Thalamus[17852]#Lung[11931,17852,05834,04470,04121,04463]#Heart[11931,17852,04470,04121,04463]#Ovary[17852]#Pancreas[11931,17852,04470,04121,04463]#Amygdala[17852]#Cerebellum[17852]#Placenta[11931,04470,04121,04463]#Subthalamic nucleus[17852]#
2.7.1.74	RL00157	deoxycytidine kinase	Thymus[00507]#T-lymphoblast[00507]#T Cell[00507]#Monocyte[00507]#Gastric mucosa[00507]#B-lymphoblast[00507]#Liver[00507]#Placenta[00507]#Colon mucosa[00507]#Brain[00507]#Muscle[00507]#Spleen[00507]#B Cell[00507]#
2.7.11.18	RL00029	myosin light chain kinase	Hippocampus[02952]#Jejunum[02952]#Fetus[02952]#Dermal microvascular endothelial cell[02952]#Liver[05953,02952]#Muscle[05953]#Brain[05953,02952]#Villus[02952]#Kidney[05953,02952]#Smooth muscle[02952]#Heart[05953,02952]#Frontal cortex[02952]#Lung[05953,02952]#Pancreas[05953,02952]#Entorhinal cortex[02952]#Placenta[05953,02952]#Umbilical vein endothelial cell[02952]#Vascular endothelium[02952]#
2.7.4.9	RL00284	deoxythymidylate kinase	Liver[01773]#Placenta[01773]#
2.7.7.1	RL00215	NMN adenylyltransferase	Thymus[16369]#Cerebrum[09786]#Hippocampus[16369,09786]#Substantia nigra[16369]#Corpus callosum[16369]#Occipital lobe[09786]#Spinal cord[16369]#Putamen[09786]#Caudate nucleus[16369,09786]#Testis[09786]#Temporal lobe[09786]#Liver[16369]#Skeletal muscle[16369,09786]#Brain[16369,09786]#Spleen[16370]#Kidney[16369,16370]#Thalamus[16369,09786]#Lung[16370]#Heart[16369,09786]#Amygdala[16369,09786]#Pancreas[16369,09786]#Cerebellum[09786]#Placenta[16369,09786,16370]#Subthalamic nucleus[16369]#Frontal lobe[09786]#
2.7.7.14	RL00016	phosphoethanolamine cytidylyltransferase	Lung[04059]#Heart[04059]#Pancreas[04059]#Skeletal muscle[04059]#Liver[04059]#Kidney[04059]#Brain[04059]#

EC code	RLEdb ID	Name	Tissue exression from HPRD
2.7.7.15	RL00015	Choline-phosphate cytidylyltransferase	Thymus[05376]#Colon[05376]#Ubiquitous[00438]#Fetus[05376]#Leukocyte[05376]#Testis[05376]#Skeletal muscle[05376]#Liver[05376]#Brain[05376]#Spleen[05376]#Reticulocyte[05376]#Prostate[05376]#Small intestine[05376]#Kidney[05376]#Lung[05376]#Heart[05376]#Ovary[05376]#Pancreas[05376]#Placenta[05376]#
3.1.1.3	RL00163	carboxyl ester lipase	Thymus[04730]#Colon[04730]#Uterus[04730]#Chief cell[09063]#Corpus luteum[04730]#Testis[04730]#Macrophage[07509,04730]#Hepatocyte[04730]#Skeletal muscle[02112,04730]#Liver[02044,02112,04730,01058]#Muscle[04730]#Mammary epithelium[04730]#Keratinocyte[04730]#Brain[02044,02112,04730]#Spleen[02112,04730]#Prostate[02112]#Small intestine[04730]#Adipose tissue[12430]#Kidney[02044,02112,04730]#Smooth muscle[04730]#Mammary gland[02044,07509]#Monocyte[07509,04730]#Lung[02044,02112,04730]#Heart[02044,02112,04730]#Ovary[04730]#Pancreas[05109,02112,07509,04730,05110,02005]#Placenta[02044,02112,04730]#Adrenal cortex[04730]#Adrenal medulla[04730]#Thyroid gland[04730]#Coronary artery[04730]#Stomach[04730,09063]#
3.1.1.4	RL00183	phospholipase a2	Colon[17858,04674,07306,10155,07499,07546,04675]#Gall bladder[01397]#Hippocampus[04673]#Lymph node[04675]#Corpus callosum[04673]#Choriodecidua[08986]#Cartilage[01397]#Fetus[04674]#Urinary bladder[10155]#Testis[17858,07306,10155,07499,04675]#Macrophage[03117]#Endocrine pancreas[10155]#Brain[17858,04673,07143,07306,07499,01397,04675]#Spleen[01396,04674,07306,07499,07546,04675]#Small intestine[17858,07306,10155,07499,07546,04675]#Chorioamnionic membrane[03117]#Thalamus[04673]#Heart[04673,07144,17858,07143,01396,07306,07499,10155,03117,04675]#Urine[01396]#Amygdala[04673]#Nasal mucosa[01396]#Mast cell[03117]#Umbilical vein endothelial cell[03117]#Thyroid gland[10155,04675]#Thymus[17858,01396,04674,07306,07499,07546,04675]#Uterus[10155]#Substantia nigra[04673]#Vulva[10155]#Spinal cord[01396,04675]#Tear[01396]#Leukocyte[17858,04674,07306,07499,07144,04675]#Intestine[01397]#Caudate nucleus[04673]#Bone marrow[04675]#Liver[17858,04673,07306,10155,07499,07144,04675]#Skeletal muscle[17858,04673,07306,10155,07499,07144,04675]#Joints[01397]#Prostate[17858,07306,10155,07499,07546,04675]#Platelet[01397]#Ki
3.1.1.7	RL00023	acetylcholinesterase	Nervous system[00010]#Thalamus[00010]#Osteoblast[00010]#Liver[00010]#Blood[00010]#Retina[00010]#Brain[00010]#Muscle[00010]#B Cell[00010]#

EC code	RLEdb ID	Name	Tissue exression from HPRD
3.1.3.11	RL00283	hexose diphosphatase	Lung[01973]#Skeletal muscle[04322]#Liver[01973]#Muscle[01973]#
3.1.3.4	RL00253	phosphatidate phosphohydrolase	Thymus[06179]#Colon[06178,06179]#Fetus[06180]#Leukocyte[06179]#Testis[06178,06179]#Skeletal muscle[06178,06179]#Liver[06178,06179]#Blood[06179]#Brain[06178,06179]#Spleen[06178,06179]#Prostate[06178,06179]#Small intestine[06178,06179]#Kidney[06178,06179]#Lung[06178,06179]#Heart[06178,06179]#Pancreas[06178,06179,06180]#Ovary[06178,06179]#Placenta[06179,06180]#
3.1.3.5	RL00111	5'-nucleotidase	Thymus[00552]#Hematopoietic stem cell[05871]#Uterus[14844]#Cerebral cortex[00552]#Glomerulus[00552]#Leukocyte[00552]#Testis[11405,14844]#Skeletal muscle[11405,11833,14844,12009]#Liver[11405,14844]#Myocardium[00552]#Brain[11405,14844,12009]#Spleen[00552]#B Cell[00552]#Intestinal epithelium[00552]#Dendritic cell[00552]#Vascular endothelial cell[00552]#Kidney[11405,14844,12009]#T Cell[00552]#Lung[11405,14844]#Heart[11405,11833,14844,12009]#Pancreas[11405,11833,14844,12009]#Cerebellum[00552]#Placenta[00552,11405,02686,11833,14844]#Lymphocyte[05871]#Eosinophil[00552]#
3.1.3.9	RL00150	Glucose-6-phosphatase	Gall bladder[01983]#Kidney[01983]#Testis[16272,01983]#Islets of Langerhans[16272]#Pancreas[16272]#Liver[01983]#Placenta[01983]#Brain[01983]#
3.1.6.2	RL00108	sulfatase	Lung[02389]#Intestine[02389]#Heart[02389]#Ovary[02389]#Liver[02389]#Placenta[02389]#Adrenal gland[02389]#Thyroid gland[02389]#Muscle[02389]#Spleen[02389]#
3.2.1.24	RL00018	alpha-mannosidase	Colon[02007]#Hippocampus[14355]#Substantia nigra[14355]#Corpus callosum[14355]#Spinal cord[14355]#Fetus[14355]#Caudate nucleus[14355]#Testis[14355]#Skeletal muscle[14355]#Liver[02007,14355]#Brain[02007,14355]#Muscle[02007]#Spleen[02007,14355]#Kidney[02007,14355]#Thalamus[14355]#Lung[02007,14355]#Heart[02007,14355]#Ovary[14355]#Pancreas[02007,14355]#Amygdala[14355]#Placenta[02007]#Cerebellum[14355]#Subthalamic nucleus[14355]#Lymphocyte[01109]#

EC code	RLEdb ID	Name	Tissue exression from HPRD
3.4.14.5	RL00156	dipeptidyl peptidase	Serum[02187]#Colon[07076,07001,02187]#Jejunum[02187]#Ubiquitous[07076,07001]#Fetus[07001]#Testis[07076,07001]#Thymocyte[02187]#Brain[07076,07001,02187]#Spleen[07076,07001,02187]#Small intestine[07076,07001]#Bronchus[02187]#Cytotrophoblast[02187]#Endometrium[02187]#Heart[07076,07001,02187]#Lymphocyte[02187]#Th(1) cell[02187]#Natural killer cell[02187]#Duodenum[02187]#Thymus[07076,07001]#Uterus[07001]#Intestine[07076]#Leukocyte[07076,07001]#Liver[07076,07001,02187]#Skeletal muscle[07076,07001,02187]#Muscle[07076]#Keratinocyte[02187]#Prostate[07076,07001]#Kidney[07076,07001,02187]#Th(2) cell[02187]#Lung[07076,07001,02187]#Ovary[07076]#Pancreas[07076,07001,02187]#Ileum[02187]#Plasma[02187]#Skin[02187]#Placenta[07076,07001,02187]#Semen[02187]#
3.4.21.5	RL00177	thrombin	Serum[01488]#Plasma[01488]#Ovary[01488]#Liver[01488]#
3.4.23.46	RL00011	Beta-secretase	Colon[07255]#Cerebral cortex[07255]#Leukocyte[07255]#Muscle fibre[07255]#Liver[07255]#Muscle[07255]#Brain[07255]#Spleen[07255]#Prostate[07255]#Cerebral neuron[07255]#Small intestine[07255]#Kidney[07255]#Lung[07255]#Heart[07255]#Ovary[07255]#Pancreas[07255]#Placenta[07255]#Thyroid gland[07255]#
4.1.1.17	RL00247	ornithine decarboxylase	Colon[01324]#Hippocampus[01324]#CNS[01324]#Spinal cord[01324]#Skin fibroblast[01324]#Leukocyte[01324]#Oesophagus[01324]#Skin[01324]#Liver[01324]#Lymphocyte[01324]#Keratinocyte[01324]#Stomach[01324]#Prostate[01324]#
4.1.1.32	RL00003	phosphoenolpyruvate carboxykinase (GTP)	Small intestine[02026,02028]#Kidney[02026,02028]#Heart[02026]#Lung[02026]#Pancreas[02026]#Liver[02026,02028]#Placenta[02026]#Brain[02026]#
4.2.1.22	RL00005	cystathionine-beta-synthase	Lung[01994]#Heart[01994]#Skin[01994]#Pancreas[01994]#Liver[01994]#Brain[01994]#
4.2.1.24	RL00281	delta-aminolevulinic acid synthase	Ovary[00504]#Liver[00504]#Red blood cell[00504]#
4.2.3.12	RL00135	pyruvoyl tetrahydropterin synthase	Dendritic cell[11840]#Platelet[11840]#T Cell[11840]#Monocyte[11840]#Macrophage[11840]#Liver[11840]#Brain[11840]#Reticulocyte[11840]#
4.4.1.1	RL00020	cystathionine-gamma-lyase	Liver[09633]#

EC code	RLEdb ID	Name	Tissue exression from HPRD
4.4.1.20	RL00217	LTC4 synthase	Thymus[02004]#Colon[02004]#Uterus[02004]#Salivary gland[02004]#Macrophage[02004]#Bone marrow[02004]#Skeletal muscle[02004]#Liver[02004]#Lymph[02004]#Alveolar macrophage[02004]#Brain[02004]#Spleen[02004]#Platelet[02004]#Kidney[02004]#Langerhans cell[02004]#Adrenal gland[02004]#Monocyte[02004]#Granulocyte[02004]#Lung[02004]# Myeloid stem cell[02004]#Skin[02004]#Epidermis[02004]#Basophil[02004]#Trachea[02004]#Placenta[02004]#Mast cell[02004]#Stomach[02004]#Pituitary gland[02004]#Eosinophil[02004]#
5.1.3.14	RL00251	n acetylglucosamine epimerase	Kidney[04825]#Heart[04825]#Lung[04825]#Pancreas[04825]#Liver[04825]#Placenta[04825]#Brain[04825]#Muscle[04825]#
5.2.1.8	RL00137	prolyl cis trans isomerase	Colon[15163,11063,05326,07606,04019,03893,08472,02795]#Nervous system[03031]#Hippocampus[01743]#Lymph node[08472,04019]#Ubiquitous[15163,11875,03194,08472,02795]#Fetus[00457,05326,11875,03194,07606,12083,02795]#Testis[15163,11063,05326,07606,04019,03893,08472,02795,12083]#Islets of Langerhans[00457,00458,11875]#Brain[00457,01741,06839,11875,08472,03893,05327,11063,15163,05326,04019,07606,02219,00459,11794,12083]#B Cell[12083]#Spleen[15163,11063,05326,07606,04019,03893,08472,02795]#Small intestine[15163,11063,05326,07606,04019,03893,08472,02795,12083]#Endometrium[08472]#Heart[15163,11063,05326,07606,04019,02219,11875,03194,08472,03893,05327,12083,11794,00459,02795]#Cerebellum[02795]#Thyroid gland[08472]#Lymphocyte[00457,00458]#Cerebrum[02795]#Thymus[15163,11063,05326,07606,04019,03893,08472,02795,12083]#Uterus[08472]#Leukocyte[15163,11063,05326,07606,04019,01741,03893,08472,11794,02795]#Intestine[04019]#Liver[01741,06839,11875,08472,03893,05327,11063,15163,05326,04019,07606,02219,02795,00459,11794,12083]#Skeletal muscle[15163,11063,05326,07606,04019,02219,11875,08472,03893,05
5.3.3.1	RL00033	beta hydroxysteroid dehydrogenase/ isomerase	Uterus[01941]#Intestine[01941]#Testis[01941]#Liver[01941]#Muscle[01941]#Brain[01941]#Prostate[01941,04920]#Epididymis[01941]#Pulmonary artery[04920]#Adipose tissue[01941]#Kidney[01941]#Adrenal gland[01941]#Mammary gland[01941,04920]#Heart[01941]#Lung[01941]#Ovary[01941]#Skin[01941,04920]#Placenta[01941,04920]#Vascular endothelium[04920]#Sebaceous gland[04920]#Pituitary gland[01941]#

EC code	RLEdb ID	Name	Tissue exression from HPRD
5.3.4.1	RL00200	protein disulfide isomerase	Serum[07504]#Hippocampus[15591]#Substantia nigra[15591]#Corpus callosum[15591]#Spinal cord[15591]#Fetus[15591]#Caudate nucleus[15591]#Testis[15591]#Islets of Langerhans[07504,07181,03625]#Skeletal muscle[15591,03625]#Liver[15591,07181,03625]#Brain[08334,15591,03625]#Spleen[15591]#Kidney[08334,15591,03625]#Thalamus[15591]#Neutrophil[06501]#Lung[08334,15591,03625]#Heart[15591,03625]#Pancreas[15591,03625,07617]#Ovary[07504,15591,07181,03625]#Amygdala[15591]#Placenta[07504,08334,07181,03625]#Cerebellum[15591]#Subthalamic nucleus[15591]#
5.4.99.7	RL00268	oxido:lanosterol cyclase/Oxidosqualene--lanosterol cyclase	Kidney[02945]#Heart[02945]#Lung[02945]#Pancreas[02945]#Liver[02945]#Skeletal muscle[02945]#Placenta[02945]#Brain[02945]#
5.5.1.4	RL00061	l myo inositol phosphate synthase	Thymus[17163]#Colon[17163]#Leukocyte[17163]#Testis[17163]#Skeletal muscle[17163]#Liver[17163]#Brain[17163]#Spleen[17163]#Prostate[17163]#Small intestine[17163]#Kidney[17163]#Lung[17163]#Heart[17163]#Pancreas[17163]#Ovary[17163]#Placenta[17163]#

EC code	RLEdb ID	Name	Tissue exression from HPRD
6.2.1.3	RL00086	arachidonyl coA synthetase	Peripheral blood cell[09190]#Colon[03845,16139,02152]#Hippocampus[09190]#Colon epithelium[02152]#Corpus callosum[09190]#Lymph node[02152]#Fetus[09190,02152]#Testis[09190,03845,16139,02152]#Red blood cell[09190]#Brain[01068,11935,09190,03845,16139,02152]#Spleen[09190,03845,16139,02152]#Small intestine[16139,02152]#Thalamus[09190]#Intestinal mucosa[16139]#Heart[01068,11935,09190,03845,16139,02152]#Amygdala[09190]#Cerebellum[09190]#Adrenal medulla[02152]#Thyroid gland[02152]#Duodenum[01068]#Thymus[03845,16139,02152]#Substantia nigra[09190]#Spinal cord[09190,02152]#Skin fibroblast[11935]#Caudate nucleus[09190]#Leukocyte[03845,16139,02152]#Intestine[03845]#Bone marrow[09190,02152]#Liver[01068,11935,09190,03845,16139,02152]#Skeletal muscle[01068,09190,16139,02152]#Muscle[03845]#Prostate[03845,16139,02152]#Villus[16139]#Kidney[01068,11935,09190,03845,16139,02152]#Adrenal gland[02152]#Lung[01068,09190,03845,16139,02152]#Ileum[02152]#Pancreas[01068,11935,09190,03845,16139,02152]#Ovary[09190,03845,02152]#Adrenal cortex[02152]#Placenta[01068,11935,03845,16139,02152]#Trachea[02152]#Subthalamic nucleus[09190]#Stomach[02152]#
6.2.1.5	RL00240	ATP-dependent citrate lyase	Thymus[06798]#Colon[06798]#Lymph node[06798]#Ubiquitous[06798]#Fetus[06798]#Leukocyte[06798]#Testis[06798]#Bone marrow[06798]#Skeletal muscle[06798]#Liver[06798]#Brain[06798]#Spleen[06798]#Prostate[06798]#Small intestine[06798]#Kidney[06798]#Heart[06798]#Ovary[06798]#Pancreas[06798]#Placenta[06798]#
6.2.1.7	RL00188	microsomal bile acid coA synthetase	Testis[04499]#Liver[04499]#
6.3.2.2	RL00234	gamma-glutamylcysteine synthetase(gamma-GCShs)	Liver[06032]#Kidney[06032]#
6.3.4.16	RL00021	Carbamyl phosphate synthetase I	Intestine[01995]#Liver[01995]#

EC code	RLEdb ID	Name	Tissue exression from HPRD
6.4.1.2	RL00242	acetyl CoA carboxylase	Thymus[01938]#Salivary gland[01938]#Testis[01938]#Skeletal muscle[01938,07044]#Liver[01938,07044]#Muscle[01938]#Brain[01938]#Prostate[01938]#Adipose tissue[01938]#Kidney[01938]#Adrenal gland[01938]#Mammary gland[01938]#Lung[01938]#Heart[01938,07044]#Pancreas[01938]#Cerebellum[01938]#Placenta[01938]#
